# PTSD Increases Risk for Hypertension Development Through PVN Activation and Vascular Dysfunction in Sprague Dawley Rats

**DOI:** 10.3390/antiox13111423

**Published:** 2024-11-20

**Authors:** Xinqian Chen, Xin Yan, Chunxiu Yu, Qing-hui Chen, Lanrong Bi, Zhiying Shan

**Affiliations:** 1 Department of Kinesiology and Integrative Physiology, Michigan Technological University, Houghton, MI 49931, USA; xinqianc@mtu.edu (X.C.); qinghuic@mtu.edu (Q.-h.C.); 2 Health Research Institute, Michigan Technological University, Houghton, MI 49931, USA; xinyan@mtu.edu (X.Y.); chunxiuy@mtu.edu (C.Y.); 3 Department of Chemistry, Michigan Technological University, Houghton, MI 49931, USA; 4 Department of Biomedical Engineering, Michigan Technological University, Houghton, MI 49931, USA

**Keywords:** posttraumatic stress disorder, single prolonged stress, paraventricular nucleus, oxidative stress, vascular dysfunction, hypertension

## Abstract

This study investigates the impact of single prolonged stress (SPS), a model of post-traumatic stress disorder (PTSD), on cardiovascular responses, hypothalamic paraventricular nucleus (PVN) activity, and vascular function to elucidate the mechanisms linking traumatic stress to hypertension. Although SPS did not directly cause chronic hypertension in male Sprague Dawley (SD) rats, it induced acute but transient increases in blood pressure and heart rate and significantly altered the expression of hypertension-associated genes, such as vasopressin, angiotensin II type 1 receptor (AT1R), and FOSL1 in the PVN. Notably, mitochondrial reactive oxygen species (mtROS) were predominantly elevated in the pre-autonomic regions of the PVN, colocalizing with AT1R- and FOSL1-expressing cells, suggesting that oxidative stress may amplify sympathetic activation and stress responses. SPS also increased mRNA levels of pro-inflammatory cytokines (TNFα and IL1β) and inducible nitric oxide synthase (iNOS) in the aorta, and impaired vascular reactivity to vasoconstrictor and vasodilator stimuli, reflecting compromised vascular function. These findings suggest that SPS-sensitize neuroendocrine, autonomic, and vascular pathways create a state of cardiovascular vulnerability that could predispose individuals to hypertension when exposed to additional stressors. Understanding these mechanisms provides critical insights into the pathophysiology of stress-related cardiovascular disorders and underscores the need for targeted therapeutic interventions that address oxidative stress and modulate altered PVN pathways to mitigate the cardiovascular impact of PTSD and related conditions.

## 1. Introduction

Post-traumatic stress disorder (PTSD) is a complex psychiatric condition associated with an increased risk of hypertension, a major risk factor for cardiovascular disease—the leading cause of death worldwide [1,2,3]. Studies, particularly among veterans, have demonstrated that individuals with PTSD are significantly more likely to develop hypertension compared to those without the disorder [4,5,6]. Despite this established association, the mechanisms linking PTSD to elevated blood pressure (BP) remain poorly understood. Understanding these mechanisms is critical, as it could inform the development of targeted interventions to prevent or mitigate the progression of hypertension and subsequent cardiovascular disease in individuals with PTSD.

The central nervous system plays a pivotal role in regulating BP and cardiovascular function by modulating sympathetic outflow and hormone levels [7,8]. Hyperactivity of sympathetic outflow and dysregulation of hormonal pathways are key factors in the development of hypertension and cardiovascular disease [9,10]. Among the brain regions involved, the hypothalamic paraventricular nucleus (PVN) is particularly important. The PVN integrates various neural and hormonal signals to modulate physiological processes, including cardiovascular regulation [11]. Two critical components within the PVN, vasopressin (AVP) and the angiotensin II type 1 receptor (AT1R), are essential in controlling BP and cardiovascular function [12,13]. AVP-producing neurons project to the posterior pituitary gland, releasing AVP into circulation to stimulate water reabsorption and vasoconstriction, thereby influencing BP. AT1R, a key component of the renin–angiotensin system (RAS), interacts with angiotensin II to regulate BP and the hypothalamic–pituitary–adrenal axis [13,14]. Dysregulation of these pathways in the PVN is hypothesized to link PTSD to cardiovascular dysfunction [15,16,17].

Oxidative stress, characterized by an imbalance between reactive oxygen species (ROS) production and antioxidant defenses, is another major contributor to hypertension [18]. Oxidative stress within the PVN has been linked to increased sympathetic nervous activity and is observed in various hypertension models [19,20,21]. Given the PVN’s involvement in stress responses, PTSD-induced anxiety may alter the expression of genes involved in BP regulation, such as AVP and AT1R, and elevate oxidative stress within the PVN, potentially contributing to hypertension.

Beyond central nervous system dysregulation, vascular dysfunction—including endothelial dysfunction and inflammation—plays a significant role in hypertension [22,23]. A key aspect of endothelial dysfunction is the reduction in nitric oxide (NO) bioavailability, primarily produced by endothelial nitric oxide synthase (eNOS). Insufficient NO impairs vasodilation, contributing to elevated BP [23]. Vascular inflammation further exacerbates this dysfunction by impairing NO production and increasing vascular resistance [22,24]. The interplay between neuroendocrine dysregulation, oxidative stress, and vascular dysfunction could form a mechanistic pathway linking PTSD to hypertension.

Given this background, we hypothesize that PTSD may contribute to the development of hypertension through the dysregulation of PVN activation and induction of vascular dysfunction. This study utilizes the single prolonged stress (SPS) paradigm, a widely accepted model of PTSD, to induce PTSD-related symptoms in normal Sprague Dawley (SD) rats [25]. Our primary objectives are to investigate whether SPS increases BP and alters the expression of key cardiovascular-related genes in the PVN, including AVP, AT1R, and Fos-like antigen 1 (FOSL1), a marker of chronic neuronal activation, and to examine the role of mitochondrial ROS (mtROS) in these processes. Additionally, we assess the impact of SPS on vascular health by evaluating vascular inflammatory cytokines, eNOS abundance, and overall vascular function.

## 2. Materials and Methods

### 2.1. Animals

Adult male SD rats were obtained from Charles River Laboratories (Wilmington, MA, USA) and utilized in our breeding colony to generate offspring. The rats were housed under controlled conditions, with an ambient temperature ranging from 20 to 24 °C and a 12 h light–dark cycle. The rats were provided ad libitum access to food and water throughout the experimental period. All experimental protocols were approved by the Institutional Animal Care and Use Committee (IACUC) at Michigan Technological University.

### 2.2. SPS Experimental Paradigm

SD rats were randomly assigned to either the control or SPS group. The SPS procedure was administered individually to each rat as described below. First, the rats were immobilized for 2 h in AIMS™ rodent restraint bag (Fisher Scientific, Waltham, MA, USA). Immediately afterward, they were subjected to a 20 min forced swim in a tank filled with water at approximately 25 °C. The tank’s depth was carefully calibrated so that the rats could not keep their noses above the water surface when their tails touched the bottom. Following the swim, a 10 min recovery period was provided. Subsequently, the rats were exposed to diethyl ether in a sealed glass container until they lost consciousness. Finally, the rats were individually housed and left undisturbed for 7 days.

### 2.3. BP Measurement

Eight-week-old male SD rats were anesthetized, and telemetry transducers (HD-S10, Data Science, St. Paul, MN, USA) were implanted into their femoral artery for BP and heart rate (HR) measurement [26]. Briefly, rats were anesthetized with isoflurane (2–3%), a catheter connected to a telemetry transmitter was inserted into the femoral artery, and the transmitter was placed subcutaneously. Following a recovery period of 1 week from the surgery, baseline recordings of mean arterial BP (MAP) and HR were obtained from conscious rats before initiating any treatment. Subsequent recordings were conducted on the initial day and at 7-day intervals post-treatment.

### 2.4. mRNA Level Measurement in the PVN and Aorta

Seven days post-SPS, animals were euthanized with an overdose of isoflurane. Subsequently, their brains and aortas were removed and stored at −80 °C until further use. The hypothalamic PVN was punched out to measure the mRNA levels of AVP, AT1R, and FOSL1. The aorta was ground into powders with liquid nitrogen. RNA isolation was performed using the RNeasy Mini kit (Qiagen, Germantown, MD, USA) following the manufacturer’s instructions. Reverse transcription of RNA (200–400 ng from each sample) was carried out to synthesize cDNA, serving as a template in Real-Time PCR assays. TaqMan primers and probes specific for AVP (Rn00690189_g1), AT1R (Rn01435427_m1), FOSL1 (Rn00564119_m1), tumor necrosis factor alpha (TNFα, Rn99999017_m1), interleukin-1 beta (IL-1β, Rn00580432_m1), interleukin 6 (IL-6, Rn01410330_m1), inducible NOS (iNOS, Rn00561646_m1), nuclear factor NF-kB subunit NF-kB1 (Rn01399583_m1), and eNOS (Rn07312037_g1) were used to measure the mRNA levels of the genes of interest, with GAPDH (Rn01775763_g1) serving as the normalization reference.

### 2.5. Immunofluorescence of AVP, AT1R, and FOSL1 in the PVN

Following SPS on day 7, animals underwent anesthesia via overdose of isoflurane, and then transcardial perfusion was carried out with cold PBS followed by paraformaldehyde (PFA) in 1 × PBS. Post-perfusion, their brains were extracted, fixed in 4% PFA overnight, and preserved in 30% sucrose in 1 × PBS until reaching the container’s bottom. Afterwards, the brains were embedded in O.C.T. compound (Sakura Finetek, Torrance, CA, USA) and cryo-sectioned into 20 µm-thick coronal sections containing PVN regions. Brain sections were then rinsed three times in PBS for 10 min each, permeabilized in cold methanol for 10 min and washed with PBS. Subsequent steps involved blocking with 5% horse serum for one hour, followed by incubation with primary antibodies including rabbit anti-FOSL1 (MBS3200399, My BioSource, San Diego, CA, USA), rabbit anti-AVP (50068, Cell Signaling, Danvers, MA, USA) or guinea pig anti-AVP (403004, Synaptic System, Goettingen, Germany), or rabbit anti-AT1R (AAR-011, Alomone Labs, Jerusalem, Israel) in PBS containing 0.5% Triton X-100 and 5% horse serum for 24 h at 4 °C. The next day, the sections underwent three additional 10 min PBS washes before incubation with secondary antibodies (Alexa Fluor 488 donkey anti-rabbit IgG or Alexa Fluor 594 goat anti-guinea pig IgG) for one hour at room temperature. After additional PBS washes, the sections were mounted in Vectorshield, and fluorescent images were captured.

### 2.6. Intracerebroventricular (ICV) Injections and mtROS Measurement

Mitochondrial-targeting fluorescent probes (MitoProbe) were employed to determine mtROS levels of brain tissues [27]. Following SPS on day 7, rats received MitoProbe injection into the right lateral ventricle, following established protocols [26]. The ICV injections were guided by stereotaxic coordinates: 0.8–0.9 mm posterior to bregma, 1.4–1.8 mm lateral to the midline, and 3.2–3.8 mm below the dural surface. Injections were administered at a flow rate of 1 µL/min using an UltraMicroPump3 (World Precision Instruments). Three hours after 10 µL MitoProbe injection (0.2 µM), an overdose of isoflurane was administered for euthanasia, followed by transcardial perfusion as described above. Immunofluorescence of AVP, AT1R, and FOSL1 was performed in brain sections containing PVN, and fluorescent images were captured using confocal microscope (Olympus FV1000, Waltham, MA, USA).

### 2.7. Quantification of mtROS Production and Immunoreactivity of AT1R, FOSL1, and AVP in the PVN Subregions

The PVN was anatomically divided into three distinct subregions: the parvocellular (Pa), magnocellular (Ma), and intermediocellular (Me) regions, as described in previous publications [28,29]. These subregions differ in both cell size and function. The Pa subregion contains smaller cells located near the third ventricle, the Ma subregion consists of larger magnocellular cells in the posterior lateral part of the PVN, and the Me region is situated between the Pa and Ma.

We quantified immunoreactivity of AVP, AT1R, and FOSL1 as well as mtROS production as described below: the total fluorescent area of AVP, AT1R, and FOSL1 in each PVN subregion was measured using ImageJ software (1.53a). After dividing the PVN into these three regions on each brain section, appropriate thresholds were set to quantify the fluorescent area in each subregion, and comparisons were made between groups. Similarly, the total fluorescence of MitoProbe in different PVN subregions was quantified by multiplying the number of MitoProbe particles by the fluorescence intensity emitted by these particles. A proper threshold was set to quantify particle numbers, and the total fluorescence was calculated based on the original fluorescent image using ImageJ software (1.53a) [30].

### 2.8. BP and HR Response to Phenylephrine (PE), Sodium Nitroprusside (SNP), and Repeated Restraint

Intraperitoneal (i.p.) injections of 100 µg PE and 200 µg SNP were administered on day 7 following SPS, with a three-hour interval between each i.p. injection. Subsequently, 40 min restraint was performed on rats individually in their home cages. Their MAP and HR were recorded one hour before the initial experiment as a baseline and one hour after each subsequent experiment.

### 2.9. Statistical Analysis

Statistical comparisons were conducted using Prism 9 (GraphPad), with all data presented as mean ± SEM. The Student’s *t*-test and two-way ANOVA test were applied to assess statistical significance. Differences in MAP and HR of each animal were calculated by subtracting baseline parameters. For experiments investigating the effect of PE, SNP, and restraint, post hoc analyses with Bonferroni multiple comparison tests were performed after establishing significant ANOVA results. The total fluorescent area was quantified using ImageJ software (1.53a). A *p*-value of less than 0.05 was considered indicative of statistical significance.

## 3. Results

### 3.1. Acute but Transient Cardiovascular Responses to SPS

**Figure 1:**
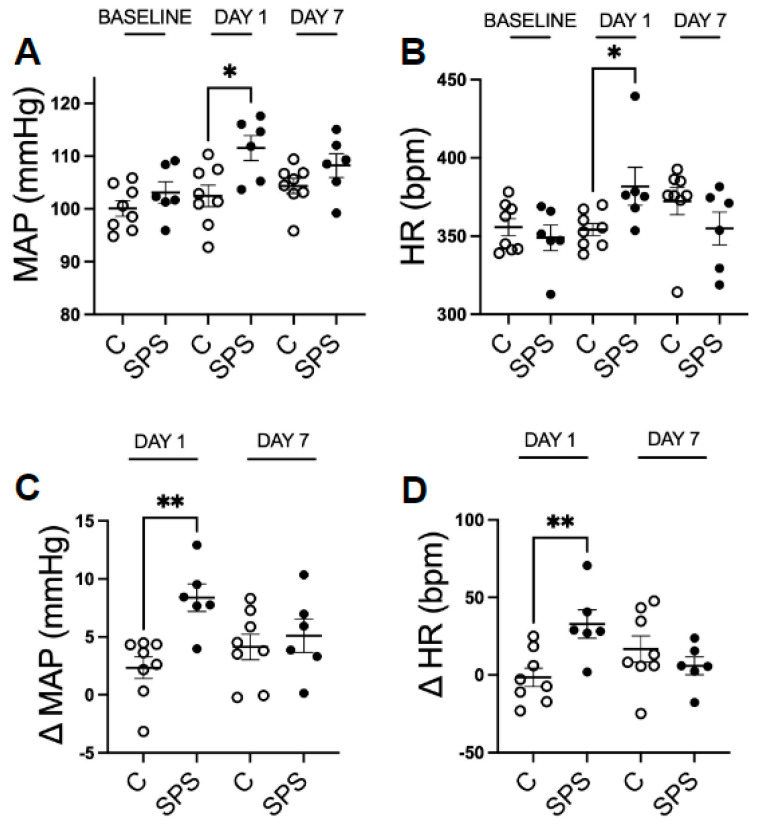
Effects of single prolonged stress (SPS) on physiological responses in male Sprague Dawley (SD) rats on day 1 and day 7 post-treatment. Mean arterial pressure (MAP, (**A**)) and heart rate (HR, (**B**)), as well as changes in MAP (ΔMAP, (**C**)) and HR (ΔHR, (**D**)) were assessed in male control SD rats (*n* = 8) and rats with SPS (*n* = 6). Data were collected at baseline, on the initial day (Day 1), and at seven days post-treatment (Day 7). Each data point represents individual values from one rat. Statistical analysis was performed using two-way unpaired Student’s *t*-test. Data are presented as mean ± SEM. * indicates *p* < 0.05, ** indicates *p* < 0.01.

The rats were randomly divided into two groups: one group received SPS treatment, while the other served as the control. Baseline MAP (Control: 100.1 ± 1.5 vs. SPS: 103.1 ± 2.0 mmHg) and HR (Control: 355.6 ± 5.5 vs. SPS: 348.9 ± 8.2 beats/min) were measured prior to treatment using a telemetry system. To evaluate the acute and longer-term effects of SPS on BP and cardiovascular function, MAP and HR were measured 5–6 h post-exposure on day 1, and again 7 days after the sensitization period. The results (Figure 1A,B) showed that on day 1, SPS rats exhibited a significant acute increase (*p* < 0.05) in both MAP (Control: 102.5 ± 2.0 vs. SPS: 111.5 ± 2.4 mmHg) and HR (Control: 354.1 ± 4.0 vs. SPS: 381.8 ± 12.1 beats/min) compared to control rats. However, by day 7, the cardiovascular responses were no longer significantly different between the SPS and control groups (MAP: Control: 104.4 ± 1.4 vs. SPS: 108.2 ± 2.3 mmHg; HR: Control: 372.4 ± 8.6 vs. SPS: 354.9 ± 10.5 beats/min), indicating that the initial cardiovascular effects of SPS were transient.

Consistently, the changes in MAP (ΔMAP) and HR (ΔHR) to their baseline in the SPS group were significantly increased on day 1 (ΔMAP: Control: 2.3 ± 0.9 vs. SPS: 8.3 ± 1.2 mmHg; ΔHR: Control: −1.5 ± 6.0 vs. SPS: 32.8 ± 9.1 beats/min, *p* < 0.01) but not on day 7 (ΔMAP: Control: 4.1 ± 1.1 vs. SPS: 5.1 ± 1.4 mmHg; ΔHR: Control: 16.7 ± 8.5 vs. SPS: 5.9 ± 5.7 beats/min), compared to control rats (Figure 1C,D). Notably, we observed an increase trend in HR in the control rats on day 7 (372.4 ± 8.6 beats/min) compared to baseline (355.6 ± 5.5 beats/min), although this difference did not reach statistical significance (*p* = 0.1). This observation suggests that factors such as social stress or subtle environmental changes may have influenced the control rats.

This finding highlights that SPS induces marked acute cardiovascular responses characterized by elevated MAP and HR immediately after exposure, but these changes do not persist beyond the acute phase. The results suggest that while SPS triggers significant immediate cardiovascular stress responses, these do not result in sustained hypertension or altered HR in the absence of additional stressors, emphasizing the need to consider both short- and long-term impacts of stress on cardiovascular health.

### 3.2. SPS Upregulates mRNA Expressions and Protein Activities of AVP, AT1R, and FOSL1 in the PVN

**Figure 2:**
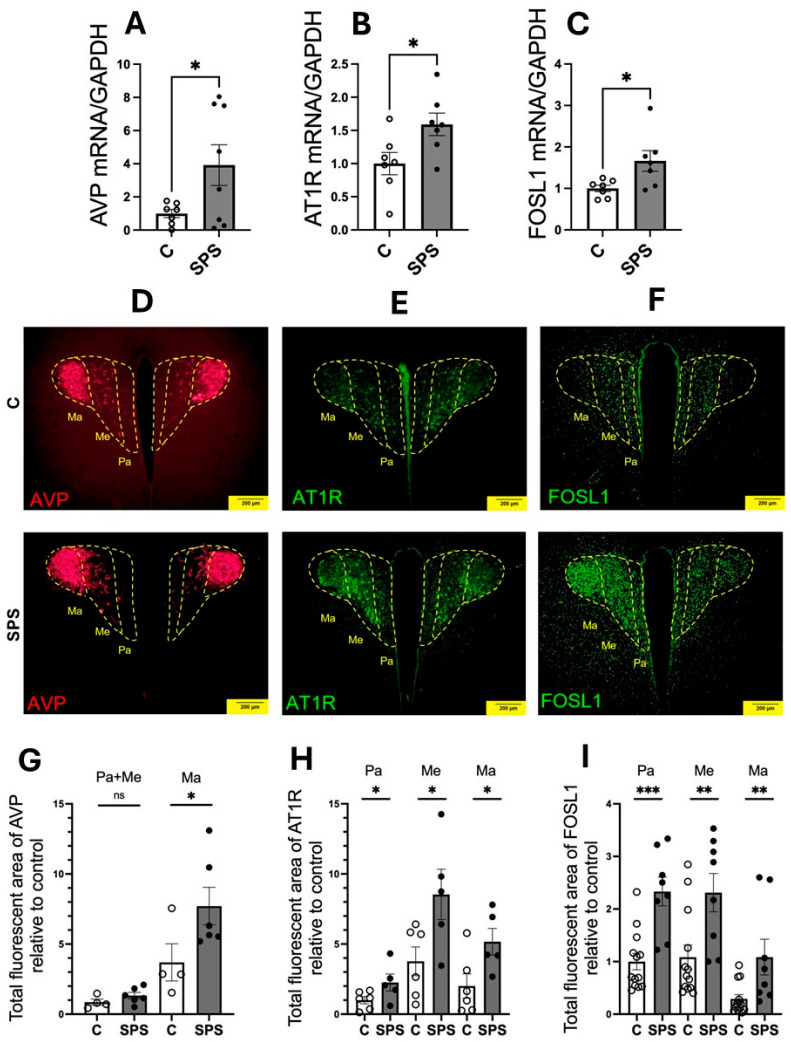
SPS increases mRNA expression and immunofluorescence of AVP, AT1R, and FOSL1 in the hypothalamic paraventricular nucleus (PVN) of male SD rats. The mRNA levels of AVP (**A**), AT1R (**B**), and FOSL1 (**C**) were measured in the PVN of male SD rats with and without SPS exposure. mRNA expression was semi-quantified using real-time PCR and normalized to GAPDH, comparing control and SPS groups. Each data point represents individual values from one rat (*n* = 7–8 per group). Statistical analysis was performed using a two-way unpaired Student’s *t*-test. (**D**–**F**) Representative fluorescent images show AVP ((**D**), red), AT1R ((**E**), green), and FOSL1 ((**F**), green) within the PVN. Scale bar = 200 µm. The total fluorescent areas of AVP (**G**), AT1R (**H**), and FOSL1 (**I**) were quantified in the parvocellular (Pa), intermediocellular (Me), and posterior magnocellular lateral (Ma) subregions of the PVN and normalized to control values. Each data point represents a microscopic view of a PVN region. Statistical analysis was conducted using a one-way unpaired Student’s *t*-test. Data are presented as mean ± SEM. * indicates *p* < 0.05, ** *p* < 0.01, *** *p* < 0.001 and ns indicates *p* > 0.05.

To assess the impact of SPS on the expression of genes involved in cardiovascular regulation within the PVN, mRNA levels of AVP, AT1R, and FOSL1 were measured in the PVN of SPS-exposed and control rats on day 7 post-treatment. The results demonstrated a significant upregulation of AVP (3.9-fold), AT1R (1.6-fold), and FOSL1 (1.6-fold) mRNA levels in SPS rats compared to controls (*p* < 0.05; Figure 2A–C). Immunofluorescence analysis further confirmed increased protein levels of AVP, AT1R, and FOSL1 in the PVN of SPS-exposed rats, indicating enhanced expression at both the mRNA and protein levels (Figure 2D–I).

The PVN is known to comprise the Pa, Me, and Ma subregions, which differ in both cell size and function [28,29]. The Pa subregion, characterized by smaller cells located near the third ventricle, is primarily involved in regulating sympathetic and cardiovascular responses. The Ma subregion, consisting of larger magnocellular cells in the posterior lateral part of the PVN, produces hormones and projects to the posterior pituitary gland, where oxytocin or vasopressin is released into the bloodstream. The Me region, situated between the Pa and Ma, is rich in pre-autonomic neurons, suggesting its role in controlling sympathetic activity and cardiovascular function. To better understand the functional significance of gene alterations in the PVN, we quantified the immunoreactivity of AVP, AT1R, and FOSL1 in different PVN subregions and compared SPS-exposed rats with controls. Our results revealed a significant 2.1-fold increase in AVP immunoreactivity specifically in the Ma region (Figure 2D,G), indicating an enhanced neuroendocrine response to SPS. However, no significant changes were observed in the pre-autonomic nuclei (Pa and Me). In contrast, both AT1R and FOSL1 showed significant increases across all PVN subregions, with particularly notable upregulation in the Pa and Me regions, which are crucial for autonomic regulation (Figure 2E,F,H,I). This regional specificity highlights the differential effects of SPS on the PVN, suggesting that SPS activates distinct subregions and enhances molecular pathways contributing to altered cardiovascular regulation.

These findings demonstrate that SPS significantly upregulates AVP, AT1R, and FOSL1 in the PVN, with enhanced protein activity in areas associated with both autonomic and neuroendocrine functions. The selective increase in AVP in the magnocellular region points to a specific neuroendocrine response, while the widespread upregulation of AT1R and FOSL1 suggests a broader activation of the PVN’s autonomic pathways. Together, these results highlight the role of SPS in modulating key molecular and regional responses within the PVN, potentially contributing to the cardiovascular dysregulation observed in conditions associated with traumatic stress.

### 3.3. SPS Elevates mtROS in AT1R- and FOSL1-Expressing Cells in the PVN

Mitochondria are a major source of ROS in cells under both normal and pathological conditions [31]. To further investigate the potential impact of SPS on brain oxidative stress, we assessed mtROS production in each PVN subregion using MitoProbe. Additionally, we examined the colocalization of mtROS with AVP, AT1R, and FOSL1 within these subregions to explore potential interactions between oxidative stress and these key regulatory molecules.

**Figure 3:**
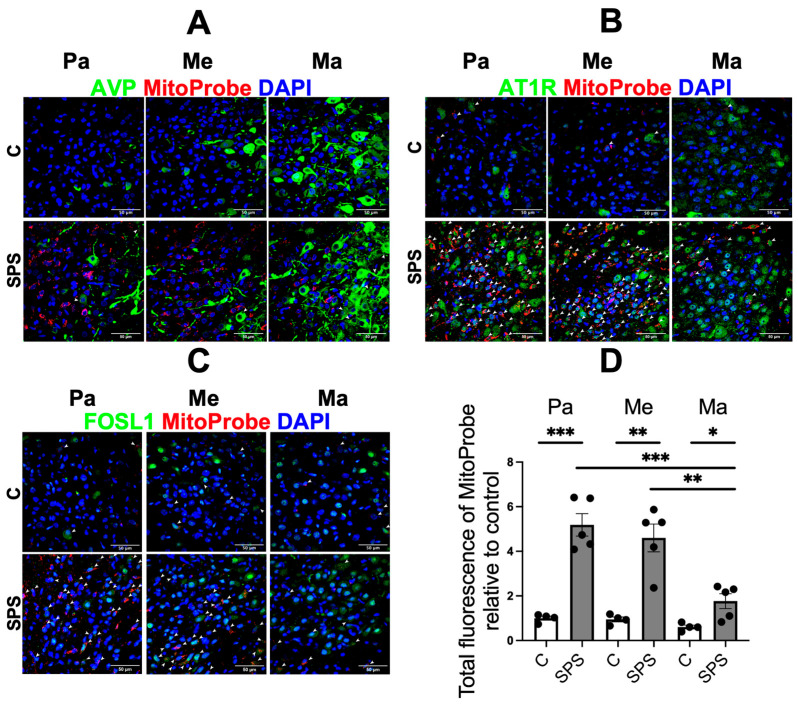
SPS increases mitochondrial reactive oxygen species (mtROS) and its colocalization with AT1R- and FOSL1-expressing cells in the PVN of SD rats. (**A**–**C**) Representative fluorescent images depicting the distribution of mtROS (in red) across PVN subdivisions, including the Pa, Me, and Ma regions. Colocalization of mtROS with AVP (**A**), AT1R (**B**), and FOSL1 (**C**) was evaluated, as indicated by white arrows. (**D**) Total fluorescence of MitoProbe in different PVN subregions was quantified and normalized to that in the Pa region of control groups. Data points represent average fluorescent intensities from multiple microscopic fields within the PVN region. The two-way Student’s unpaired *t* test was used for statistical analysis. Scale bar = 50 µm. Data are represented as mean ± SEM. * indicates *p* < 0.05, ** *p* < 0.01, *** *p* < 0.001.

Our results showed that SPS significantly increased mtROS levels in different subregions of hypothalamic PVN, as evidenced by a substantial rise in fluorescence intensity compared to controls (Figure 3D). Compared to control rats, SPS rats showed a 5.2-fold increase in the Pa region, a 5.1-fold increase in the Me region, and a 2.8-fold increase in the Ma region. This increase reflects a notable elevation in oxidative stress within the PVN of SPS-exposed rats. Analysis of subregional distribution revealed that the elevated mtROS production in SPS rats was predominantly localized in the Pa and Me subregions, with a 2.9-fold increase observed in the Pa compared to the Ma, and a 2.6-fold increase in the Me compared to the Ma.

Notably, the most pronounced mtROS enrichment occurred in cells expressing AT1R and FOSL1 within the Pa and Me subregions (Figure 3B,C). This colocalization suggests that SPS-induced oxidative stress may specifically generate in the autonomic regulatory regions of the PVN, potentially contributing to dysregulated cardiovascular responses. In contrast, the colocalization of mtROS with AVP-expressing cells was less prominent, indicating a differential impact of SPS on various molecular pathways within the PVN (Figure 3A). The elevation of mtROS in AT1R- and FOSL1-expressing cells highlights the importance of oxidative stress in the molecular and cellular alterations within the PVN following traumatic stress, providing a potential mechanistic link to the cardiovascular changes observed in PTSD and related disorders.

### 3.4. SPS Elevates mRNA Expression of Pro-Inflammatory Cytokines and Nitric Oxide Synthases in the Aorta of SD Rats

**Figure 4:**
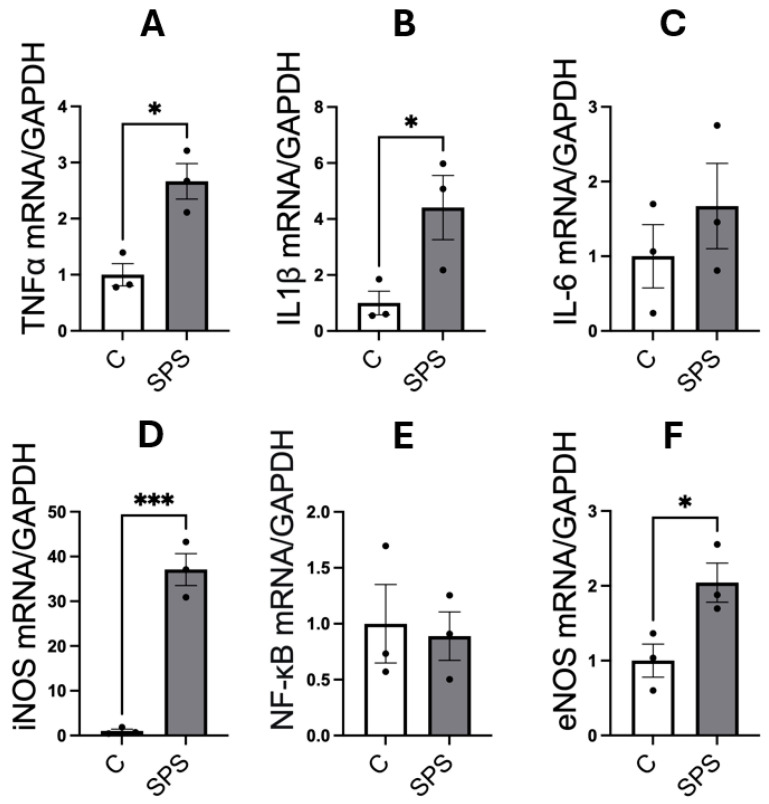
SPS increases mRNA expression of pro-inflammatory cytokines and nitric oxide synthases in the aorta of male SD rats. The mRNA levels of TNFα (**A**), IL-1β (**B**), IL-6 (**C**), iNOS (**D**), NF-κB (**E**), and eNOS (**F**) were measured in the aorta of male SD rats with and without SPS exposure. Gene expression was quantified using real-time PCR, with mRNA levels normalized to GAPDH. Statistical analysis was performed using a two-way unpaired Student’s *t*-test. Each data point represents an individual rat (*n* = 3 per group). Data are presented as mean ± SEM. Statistical significance is indicated as follows: * *p* < 0.05, *** *p* < 0.001.

Figure 4 illustrates the impact of SPS on the mRNA expression of pro-inflammatory cytokines and NOS in the aorta of male SD rats. The analysis showed that SPS significantly increased mRNA levels of the pro-inflammatory cytokines TNFα (2.6-fold, *p* < 0.05) and IL-1β (4.4-fold, *p* < 0.05) compared to controls (Figure 4A,B). A dramatic 37-fold elevation in iNOS mRNA expression was also observed in the SPS group compared to controls (*p* < 0.001; Figure 4D). However, mRNA levels of IL-6 and NF-κB were not significantly altered between SPS and control groups (Figure 4C,E). Additionally, SPS led to a significant 2-fold increase in eNOS mRNA expression compared to controls (*p* < 0.05; Figure 4F), suggesting potential changes in endothelial function.

These results indicate that SPS strongly upregulates vascular inflammatory cytokines, including TNFα, IL-1β, and particularly iNOS, which may contribute to an inflammatory environment and responses. The pronounced elevation of these cytokines, alongside iNOS, suggests that SPS potentially contribute to endothelial damage and dysfunction. The absence of significant changes in IL-6 and NF-κB may point to a selective activation of inflammatory pathways rather than a generalized inflammatory response.

These findings suggest that SPS-induced inflammatory cytokines in the aorta contribute to vascular dysfunction, highlighting a potential link between traumatic stress and the development of cardiovascular diseases. The distinct upregulation of pro-inflammatory cytokines and NOS underscores the role of SPS in driving vascular pathology, providing insights into the mechanisms by which chronic stress may predispose individuals to cardiovascular complications.

### 3.5. SPS Attenuates Cardiovascular Responses to Vasoconstrictive, Vasodilatory, and Repeated Restraint Stress in Rats

**Figure 5:**
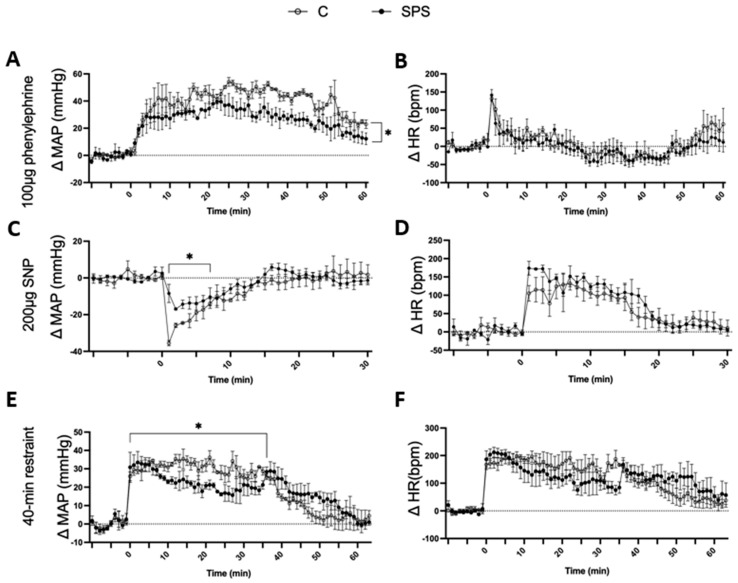
Effects of phenylephrine (PE), sodium nitroprusside (SNP), and 40 min restraint on male SD rats with and without SPS. (**A**–**D**) depict the cardiovascular responses of SPS and control rats (*n* = 3 per group) 7 days post-SPS. Rats were administered 100 μg of PE, a vasoconstrictor, and 200 μg of SNP, a vasodilator, via intraperitoneal (i.p.) injection. MAP and HR were measured before, during, and after each injection, with a minimum interval of 3 h between treatments. (**E**,**F**) show the responses to a 40 min restraint stress conducted 7 days post-SPS. Baseline BP and HR were recorded prior to the start of the restraint. A two-way ANOVA test was used to assess differences between SPS and control groups. Data are presented as mean ± SEM. * indicates *p* < 0.05.

SPS rats showed a significantly attenuated MAP response to PE compared to controls. After PE injection, SPS rats exhibited a gradual increase in ΔMAP, but the response was significantly lower (Δ23.01 ± 1.4 mmHg) compared to control rats (Δ33.55 ± 2.0 mmHg, *p* < 0.05; Figure 5A). There was no significant difference in HR response between the two groups following PE administration (Figure 5B). Similarly, SPS rats displayed a reduced MAP response to SNP. Within the first 3 min of SNP injection, ΔMAP decreased in both groups; however, SPS rats had a significantly attenuated response (Δ−12.87 ± 1.0 mmHg) compared to controls (Δ−22.75 ± 2.7 mmHg, *p* < 0.05; Figure 5C). The HR response to SNP was comparable between the two groups, showing no significant differences (Figure 5D).

The effects of repeated restraint stress on cardiovascular response were also assessed. During the 40 min restraint, SPS rats had a significantly lower ΔMAP (23 ± 0.9 mmHg) compared to controls (30.59 ± 0.5 mmHg, *p* < 0.05; Figure 5E), even though both groups showed an increase in ΔMAP from their baseline levels. After restraint, the MAP of SPS rats remained elevated and returned to baseline levels more slowly than controls (SPS: 15.32 ± 1.6 mmHg vs. Control: 9.2 ± 1.5 mmHg, *p* < 0.05). The HR response remained similar across both groups throughout the restraint period (Figure 5F).

These results indicate that SPS impairs cardiovascular reactivity to both vasoconstrictive and vasodilatory stimuli as well as to repeated restraint stress. The attenuated MAP responses to PE and SNP suggest that SPS compromises vascular responsiveness, potentially due to altered autonomic regulation or impaired vascular smooth muscle function. The diminished MAP response during and after restraint stress further supports the idea that SPS disrupts adaptive cardiovascular responses to acute stressors. These findings highlight the potential role of SPS in contributing to vascular dysfunction and reduced cardiovascular adaptability in the face of acute and repeated stress, which could have implications for understanding the cardiovascular impacts of chronic stress exposure in clinical settings.

## 4. Discussion

The present study aimed to investigate the effects of SPS on cardiovascular responses and vascular function, with a focus on elucidating the mechanisms through which stress contributes to hypertension. Our major findings indicate that SPS elicits acute but not sustained changes in BP and HR, elevates hypertension-associated genes’ expressions and mtROS levels in the PVN, and increases vascular inflammatory cytokines as well as impaired vascular reactivity. These results suggest that traumatic stress may contribute to the progression of hypertension through persistent dysregulation of the PVN and vascular system.

Our data show that SPS led to heightened BP and HR responses on the day of stress exposure, reflecting an immediate cardiovascular response that aligns with previous studies demonstrating transient cardiovascular effects of acute stress [32,33,34,35]. However, these changes were not sustained beyond the sensitization period, indicating that SPS alone does not induce chronic cardiovascular alterations in healthy rats.

Interestingly, non-stressed control rats showed an increasing trend in HR on day 7 compared to baseline, though this difference was not statistically significant. A possible explanation is that stressful experiences can be transmitted through social and environmental cues [36]. Although the control rats were not directly exposed to stress treatment, both control and SPS rats were housed in the same room during our recordings. The HR increase observed on day 7, but not on day 1, may be due to the more pronounced behavioral changes in SPS-exposed rats after 7 days rather than within the first 24 h [37]. The control rats likely sensed and responded to the stress environment created by the SPS rats, leading to the HR increase.

This finding, combined with previous research showing that rodent models of PTSD exhibit increased susceptibility to Ang II-induced hypertension [38,39], has led us to hypothesize that while SPS does not directly cause long-term hypertension, it may prime the cardiovascular system for heightened reactivity to subsequent stressors, thereby predisposing individuals to hypertension development. To explore this, we compared the expression of hypertension-related genes including AVP, AT1R, and FOSL1, along with the oxidative stress marker mtROS, in the PVN between SPS and control rats.

Our findings demonstrate that SPS significantly alters the expression of AVP, AT1R, and FOSL1, at both mRNA and protein levels (Figure 2). Notably, increased AVP expression in the magnocellular neurons suggests an enhanced neuroendocrine response that could lead to elevated AVP secretion, promoting water retention and vasoconstriction, both of which are associated with increasing BP [12]. We also observed an upregulation of AT1R and FOSL1 throughout the PVN region. PVN AT1R’s involvement in neurosecretion and autonomic control [13,14], coupled with the sustained activation of PVN neurons indicated by increased FOSL1, suggest that they may contribute to the heightened sympathetic nerve activity observed in PTSD and other stress-related conditions [40].

Another key aspect of our findings is the significant increase in mtROS levels within the PVN following SPS. ROS produced by the mitochondria are primarily byproducts of the electron transport chain during oxidative phosphorylation [31]. Additionally, it is important to highlight that NAPDH oxidase 4 recently has been identified to locate on the mitochondria and contribute to mtROS production [41,42]. It is possible that NOX4 along with ROS generated from electron transport chains may act synergistically to enhance oxidative stress in the PVN, contributing to the development of hypertension.

Our data further indicated that SPS induced mtROS particularly in the Pa and Me regions (Figure 3). The colocalization of mtROS with AT1R- and FOSL1-expressing cells suggests that oxidative stress may play a critical role in amplifying sympathetic outflow and stress responses. This supports the notion that AT1R signaling can drive oxidative stress, which in turn enhances neuronal excitation and sympathetic nerve activity [43,44,45]. The preferential localization of mtROS in autonomic subregions may point to the selective vulnerability of sympathetic pathways to oxidative stress, which needs to be further investigated.

SPS also induced significant changes in vascular function, characterized by increased expression of pro-inflammatory cytokines, including TNFα and IL-1β, in the aorta (Figure 4). These inflammatory mediators are known to impair endothelial function, fostering vascular inflammation and contributing to vascular dysfunction [22,46]. Notably, our results demonstrated significant increases in mRNA levels of both eNOS (2-fold) and iNOS (37-fold) in the aorta of SPS rats compared to controls. Both enzymes produce NO, a crucial signaling molecule that regulates blood vessel tone and BP. Under normal conditions, NO is primarily produced by endothelial cells via eNOS, facilitating vasodilation by relaxing smooth muscle cells in blood vessel walls, which lowers BP. However, it should be noted that eNOS can become uncoupled, leading to loss of its enzymatic activity and producing superoxide instead of NO [47]. This shift contributes to oxidative stress, exacerbating vascular dysfunction. In contrast, iNOS is typically induced by inflammatory stimuli and produces larger amounts of NO [48]. Although NO generally promotes vasodilation, its reactive nature means that excessive NO can lead to the formation of peroxynitrite, a reactive nitrogen species that causes oxidative stress [49]. This oxidative stress can damage endothelial cells, impair their ability to relax, and foster vascular inflammation. As a result, blood vessel integrity is compromised, leading to increased vascular resistance, potentially contributing to hypertension and cardiovascular disease. Given the dramatic increase in iNOS expression levels compared to other factors, we speculate that iNOS may be the major contributor to NO and vascular dysfunction in this context. Consistently, impaired vascular responses were observed in SPS rats during exposure to the vasoconstrictor PE and the vasodilator SNP (Figure 5). The reduced MAP responses suggest that SPS disrupts normal vascular function, impairing the ability of blood vessels to constrict or dilate effectively. This impaired reactivity, combined with increased oxidative stress and inflammatory cytokines, highlights a potential mechanism by which chronic stress exposure may predispose individuals to cardiovascular diseases, including hypertension.

**Figure 6:**
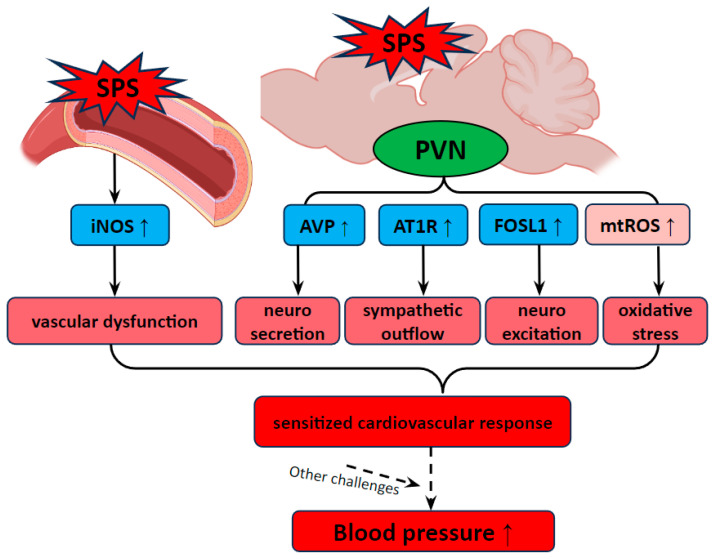
Hypothesized mechanisms of PTSD-related hypertension involving PVN activation and vascular dysfunction. Traumatic stress increases the levels of AVP, AT1R, FOSL1, and mtROS in the PVN, reflecting enhanced neurosecretory activity, heightened neuroexcitation, and increased oxidative stress within this brain region (illustrated by solid arrows). These alterations, coupled with iNOS-induced vascular dysfunction, sensitize the cardiovascular system, making it more reactive to additional stressors and contributing to the development and progression of hypertension (depicted by dashed arrows).

Our findings suggest a complex interplay between neuroendocrine and autonomic dysregulation within the PVN, amplified by oxidative stress and inflammatory cytokines in both neural and vascular systems, as a key mechanism underlying PTSD-related hypertension (Figure 6). SPS-induced increases in mtROS as well as alterations in gene expression within the PVN and aorta may enhance sympathetic outflow and disrupt vascular homeostasis, creating a state of cardiovascular vulnerability that may lead to hypertension under additional stress conditions. This hypothesized mechanism underscores the potential for traumatic stress to serve as a critical trigger for hypertension, particularly when combined with other cardiovascular stressors. Future research should focus on exploring therapeutic strategies targeting these stress-induced pathways to mitigate the long-term cardiovascular impact of PTSD and related disorders.

## 5. Conclusions

This study demonstrates that SPS induces acute but transient increases in BP and HR, disrupts the expression of hypertension-related genes in the PVN, elevates mtROS levels, elevates vascular inflammatory cytokines, and impairs vascular reactivity. Although SPS does not directly cause chronic high BP, it induces persistent changes in the PVN and vascular system that may heighten susceptibility to hypertension and cardiovascular dysfunction when exposed to additional stressors. The findings emphasize the crucial role of oxidative stress, particularly mtROS, in driving sympathetic activation and stress responses, linking neuroendocrine, autonomic, and vascular dysregulation as key mechanisms underlying the cardiovascular effects of traumatic stress.

Clinically, these results suggest that traumatic stress may predispose individuals to cardiovascular diseases, especially under further stress exposure. Understanding these pathways provides critical insights into the pathophysiology of stress-related cardiovascular disorders such as PTSD, highlighting the need for targeted therapeutic strategies that address oxidative stress and modulate altered PVN pathways. Interventions that reduce oxidative stress, regulate neuroendocrine and autonomic dysfunction, or restore vascular health could lower the risk of cardiovascular complications in individuals exposed to chronic or traumatic stress, ultimately improving clinical outcomes in this vulnerable population. Future research should focus on developing and testing such targeted approaches to protect cardiovascular health in those affected by stress-related disorders.

## 6. Limitations

This study provides valuable insights into the cardiovascular effects of SPS, particularly focusing on PVN dysregulation, oxidative stress, and vascular dysfunction. However, several limitations should be noted.

First, the research was conducted on healthy male SD rats, which limits the generalizability of the findings to other populations, such as females and individuals with pre-existing health conditions. Future studies should include both sexes and models with underlying conditions to enhance applicability. Additionally, the study only examined effects within 7 days post-stress, leaving the long-term cardiovascular impact of SPS uncertain. Lon-term studies are necessary to determine whether the observed molecular changes lead to lasting cardiovascular dysfunction.

Second, our findings show a significant increase in mtROS production in the Pa and Me subregions, particularly in AT1R- and FOSL1-expressing cells, indicating a heightened vulnerability to oxidative stress following SPS. While this suggests a potential role for oxidative stress in modulating sympathetic pathways, we acknowledge that our data do not directly demonstrate increased peripheral sympathetic activity, as we did not measure sympathetic outflow or autonomic function. Future studies should explore the role of mtROS in regulating peripheral sympathetic activity and its contribution to cardiovascular dysfunction in response to stress.

Third, our data suggest that SPS is linked to heightened levels of inflammatory cytokines in the aorta; however, they do not provide direct evidence of vascular inflammation. Future research should aim to directly assess endothelial and vascular inflammation markers to establish a clearer link between these cytokines and the onset of vascular inflammation. Investigating additional markers, such as endothelial adhesion molecules or inflammatory signaling pathways within vascular tissues, would help elucidate whether these elevated cytokines indeed lead to inflammatory changes in the vasculature.

Fourth, in the experiment testing blood vessel function in response to vasoconstrictive and vasodilatory stimuli, PE and SNP should ideally be administered to conscious rats via a pre-implanted catheter directly into the bloodstream. However, i.p. injections were used in this study, which may have delayed drug absorption and caused additional stress, potentially affecting the BP response. Despite this, since all rats received the same treatment, the observations remain valuable for evaluating vascular function. In addition, SNP is generally considered as an endothelium-independent vasodilator, as this substance directly releases NO upon reduction, targeting vascular smooth muscle cells to induce vasodilation [50]. Similarly, PE is an alpha-1 adrenergic receptor agonist, working as a vasoconstrictor to evaluate smooth muscle contractility [51]. Although our focus was on smooth muscle function, it is important to note that endothelial dysfunction is a key contributor to cardiovascular diseases. Assessing endothelial function using an endothelium-dependent vasodilator would provide deeper insights into the effects of SPS on endothelial health and NO production.

Finally, regarding the potential mechanisms contributing to impaired vascular function, although we measured eNOS mRNA levels, we did not evaluate its enzymatic activity. Further research is needed to explore eNOS function and regulation under stress conditions, specifically its activity in vascular impairment.

## Data Availability

The data presented in this study are available on request from the corresponding author due to privacy.
